# Idiopathic recurrent ischemic priapism: a review of current literature and an algorithmic approach to evaluation and management

**DOI:** 10.1186/s12610-024-00237-y

**Published:** 2024-12-04

**Authors:** Naim Yarak, Joey El Khoury, Patrick Coloby, Stéphane Bart, Maher Abdessater

**Affiliations:** NOVO Hospital, Paris, Pontoise France

**Keywords:** Stuttering priapism, Idiopathic priapism, Ischemic priapism, Pathophysiology, Management Algorithm, Therapeutic agents, Priapisme saccadé, Priapisme idiopathique, Priapisme ischémique, Physiopathologie, Algorithme de Prise en Charge, Moyens thérapeutiques

## Abstract

**Background:**

Stuttering priapism is characterized by recurrent, self-limited episodes of penile erection lasting from a few minutes to a maximum of three hours, often resolving spontaneously. These episodes can occur with or without sexual stimulation. If not treated promptly and effectively, stuttering priapism can severely impact a patient’s quality of life, leading to significant psychological distress and anxiety related to sexual performance. Although it has been associated with various hematological disorders and pharmacological treatments, many cases of stuttering priapism remain idiopathic, meaning they have no identifiable cause. Currently, no conclusive randomized clinical trials exist on the management of idiopathic stuttering priapism. This study aims to review the existing literature on the pathophysiology and management of idiopathic stuttering priapism and propose an algorithm to assist physicians in its evaluation and treatment.

**Results:**

A systematic literature review was conducted using the PubMed database, focusing on the terms “idiopathic,” “stuttering,” “ischemic,” and “priapism.” The search identified 23 relevant references published between 1991 and 2022. The selection and analysis of these studies adhered to the PRISMA (Preferred Reporting Items for Systematic Reviews and Meta-Analyses) guidelines, and results were described qualitatively. Recent research into the effectiveness, sustainability, tolerability, and side effects of various treatments for idiopathic stuttering priapism has enhanced the understanding of its underlying molecular mechanisms. Various treatments, targeting different mechanisms, have been identified that can potentially reduce the frequency and severity of episodes and improve patient outcomes.

**Conclusion:**

Current research predominantly addresses the acute treatment of idiopathic stuttering priapism rather than strategies to alter the disease’s overall course. The limited number of treatment reviews, case reports, and the low level of evidence available, combined with the absence of randomized clinical trials, prevent the establishment of a consensus on treatment protocols. As a result, idiopathic stuttering priapism remains under-recognized and under-treated. This review proposes a management framework to help clinicians access and apply the available literature effectively, minimizing the reliance on extensive case reports and review articles.

## Résumé

**Contexte:**

Le priapisme saccadé se caractérise par des épisodes récurrents et auto-limités d’érection pénienne d’une durée de quelques minutes à un maximum de trois heures, se résolvant souvent spontanément. Ces épisodes peuvent survenir avec ou sans stimulation sexuelle. S’il n’est pas traité rapidement et efficacement, le priapisme peut avoir un impact grave sur la qualité de vie d’un patient, entraînant une détresse psychologique et une anxiété importantes liées à la performance sexuelle. Bien qu’ils aient été associés à divers troubles hématologiques et traitements pharmacologiques, de nombreux cas de priapisme saccadé restent idiopathiques, ce qui signifie qu’ils n’ont pas de cause identifiable. À l’heure actuelle, il n’existe aucun essai clinique randomisé concluant concernant la prise en charge du priapisme saccadé idiopathique. Cette étude vise à passer en revue la littérature existante sur la physiopathologie et la prise en charge du priapisme idiopathique, et à proposer un algorithme pour aider les médecins dans son évaluation et son traitement.

**Résultats:**

Une revue systématique de la littérature a été réalisée à l’aide de la base de données PubMed, en se concentrant sur les termes « idiopathique », «saccadé », « ischémique » et « priapisme ». La recherche a permis d’identifier 23 références pertinentes publiées entre 1991 et 2022. La sélection et l’analyse de ces études ont respecté les directives PRISMA, et les résultats ont été décrits qualitativement. Des recherches récentes sur l’efficacité, la durabilité, la tolérabilité et les effets secondaires de divers traitements du priapisme idiopathique ont amélioré la compréhension de ses mécanismes moléculaires sous-jacents. Divers traitements, ciblant différents mécanismes, ont été identifiés qui peuvent potentiellement réduire la fréquence et la gravité des épisodes et améliorer les résultats pour les patients.

**Conclusion:**

Les recherches actuelles portent principalement sur le traitement aigu du priapisme idiopathique saccadé plutôt que sur les stratégies visant à modifier l’évolution globale de la maladie. Le nombre limité de revues sur les traitements, de cas cliniques, et le faible niveau de preuves disponibles, associés à l’absence d’essais cliniques randomisés, empêchent l’établissement d’un consensus sur les protocoles de traitement. En conséquence, le priapisme idiopathique reste sous-reconnu et sous-traité. Cette revue propose un cadre de gestion pour aider les cliniciens à accéder à la littérature disponible et à l’appliquer efficacement, en minimisant le recours à des cas cliniques détaillés et à des articles de revue.

## Introduction

The term “priapism” took its origin from Priapus who was revered as the Greek god of fertility, gardening, and lust. He is pictured with a massive phallus, a symbol of male power [1]. Priapism is best defined as a prolonged, persistent, and sometimes painful erection, which continues beyond or is even not associated with sexual stimulation. It represents such a disorder in erection and detumescence mechanisms and physiology, where typically only the corpora cavernosa are affected, while the corpus spongiosum and glans remain uninvolved [2]. Episodes lasting longer than 24–36 h or those occurring repeatedly over time may necessitate repeated treatments and subsequently may predispose patients to acquire permanent erectile dysfunction to corporal tissue ischemia and fibrosis [3]. The condition is usually divided into two major categories, ischemic (low flow, veno-occlusive) and nonischemic (high flow, arterial) priapism [4]. A third category, initially dubbed acute transitory attacks in 1914, and then “stuttering priapism”, especially in sickle-cell disease patients, designates recurrent episodes of ischemic priapism incidents which are of varying duration but are usually self-limited. The duration and incidence of these episodes can intensify, resulting in acute major ischemic priapism events requiring urgent treatment [5]. The majority of cases are idiopathic, while 21% were related to alcohol or drug use or abuse, 12% are associated with perineal trauma and 11% are linked with SCD [6]. The aim of the management strategy of stuttering priapism is not only the swift and superlative management of any acute prolonged attack to maximally maintain the erectile function but also addressing the underlying pathophysiological mechanisms to implement proactive approaches aiming to prevent the recurrence of the episodes which represents in itself the ultimate goal of the treatment [7]. The current standard of care for acute ischemic attacks for stuttering priapism in patients who experience erections lasting longer than 4 h is mechanical decompression through reactive surgical blood draining procedures with injection of sympathomimetic agents, which restores normal penile blood flow. However, this procedure is often unable to preserve erectile function thus advocating the understanding that the problem is not merely a matter of intravascular occlusion but rather constitutes a disorder of the regulatory biology of penile erection. Accordingly, following the new paramount paradigm discussing the mechanisms of the deregulatory erection physiology implicated in priapism regardless of the etiology, the relatively young age of initial onset as well as the calamitous irreversible consequences of priapism, a conceivable groundwork was instituted to effectively formulate prophylactic therapeutic options for stuttering priapism in the near future, particularly to those who are predisposed to recurrences [7, 8]. 

This article aims to review the current knowledge about pathophysiology and the management of the idiopathic entity of stuttering priapism to suggest an algorithm that can aid physicians in evaluating and managing this condition.

## Materials and methods

### Search and selection strategies

A literature review was conducted by searching PubMed using the following Medical Subject Headings (MeSH) terms: idiopathic, ischemic, stuttering, and priapism. Moreover, alternate spellings and all derivates were taken into consideration. The reference lists of the articles were also reviewed. This was done to ensure the comprehensive inclusion of articles related to the management of priapism. The review process followed PRISMA guidelines.

### Data collection process and data items

Extracted data on the evaluation or management of stuttering priapism cases were classified in an Excel sheet that was examined by two researchers for eligibility.

## Results

**Fig. 1:**
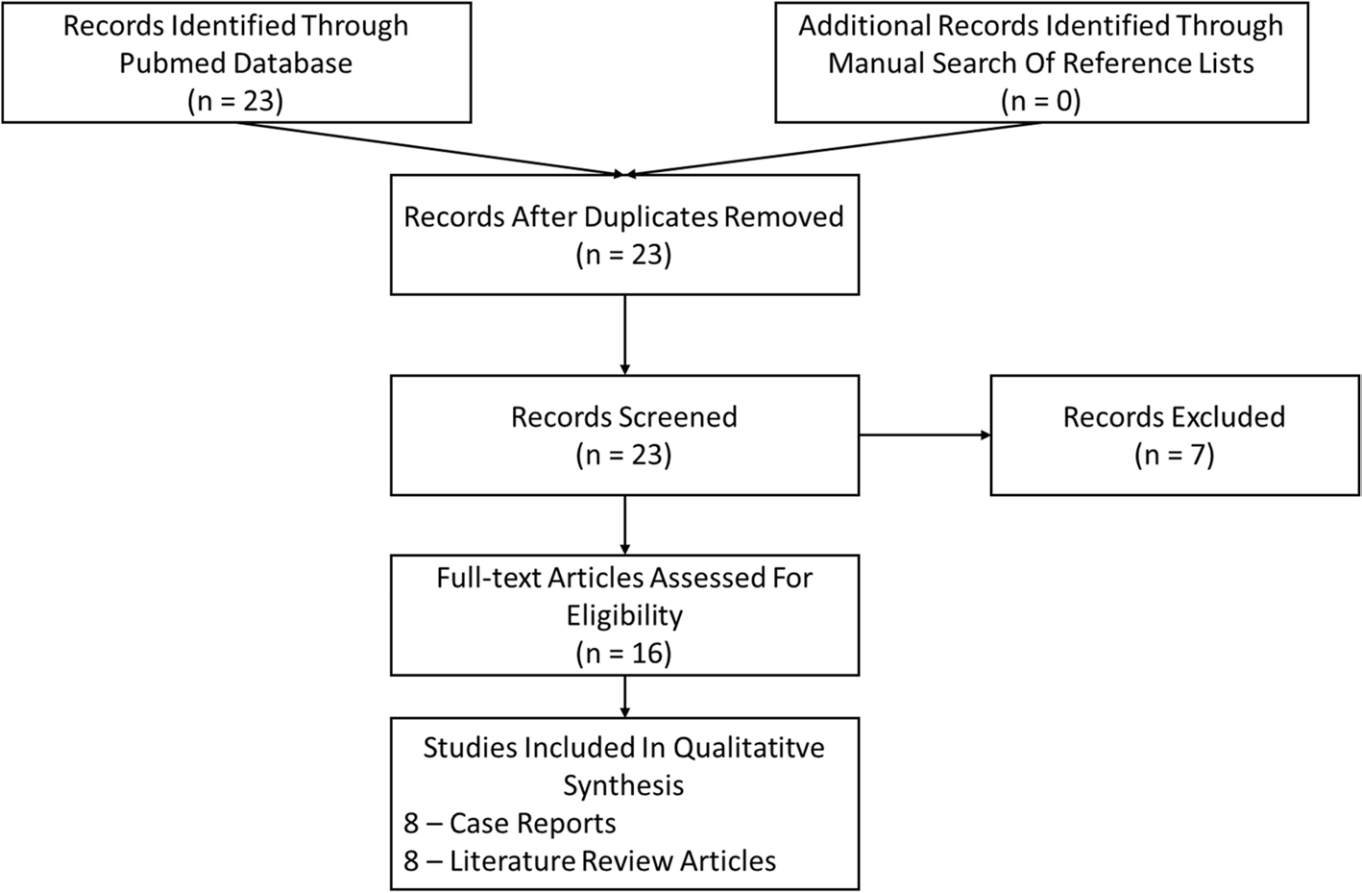
PRISMA flow chart of the article selection process. Legend : The PRISMA diagram details our search and selection process applied during the overview

The search identified 23 articles published between 1991 and 2022, of which 16 were relevant to our subject. The selection of relevant articles is displayed in a PRISMA flow diagram (Fig. 1)*.*

## Discussion

### Pathophysiology

The basic pathophysiology of stuttering priapism is not fully understood. In vivo and in vitro studies suggest that the underlying mechanisms involve an imbalance between vasorelaxant and vasoconstrictive processes responsible for erection and detumescence. Recurrence triggers are multifactorial, including changes in the corpus cavernosum microenvironment, alterations in central and peripheral neuronal systems, smooth muscle contractility issues, downregulation of adrenoreceptors, abnormal neurotransmitter regulation, and scarring of intra-cavernous venules [4, 9–11]. In priapism, a compartment-like syndrome develops due to increased pressure and reduced arterial inflow in the corpora cavernosa, leading to a hypoxic state and acidic metabolic product accumulation within 4 h. By 12 h, trabecular interstitial edema occurs, and by 24 h, thrombi fill the sinusoidal spaces, causing necrosis and smooth muscle cells potentially transforming into fibroblast-like cells. In contrast, high-flow non-ischemic priapism does not cause tissue damage because the penis maintains a high oxygenated status and retains erection potential [12–15]. In fact, smooth muscle tone is crucial for the penile response to erectile stimuli, influenced by hormones, neuroeffectors, signal transduction systems, vasoactive substances, and cellular and molecular factors. In stuttering ischemic priapism, the key issue is the disruption of smooth muscle tone regulation by the autonomic nervous system, involving agents like acetylcholine/NO/cGMP/PKG, norepinephrine, and the RhoA/Rho-kinase system. This imbalance, triggered by factors such as drugs, hematological dyscrasias, and other idiopathic causes, affects inflammation, vascular reactivity, cellular adhesion, and coagulation [10, 16–18]. In detail, and on a mechanical level, ISP is associated with veno-occlusion in the corpus cavernosum sinusoids due to erythrocyte sludging. Recent studies suggest that a key molecular factor in ISP is deficient endothelial nitric oxide (NO) synthesis and abnormal NO activity in the penile tissue, particularly in the cavernous endothelium. This disruption leads to the downregulation of downstream effectors, such as cGMP-specific protein kinase I, causing defective PDE5 regulatory function and disturbed vascular homeostasis. Neuronal NO also plays a role in inducing erections. During sexual activity, cyclic guanosine monophosphate (cGMP) accumulates in the corpora cavernosa, leading to prolonged and uncontrolled smooth muscle relaxation due to unchecked cGMP production and reduced PDE5 regulation. It is speculated that ISP may be caused by damage to the neurological and endothelial mechanisms responsible for detumescence, resulting from ischemia [4, 19, 20]. 

The RhoA and Rho-kinase signaling pathway, which normally induces penile vasoconstriction, is reduced in priapism compared to its increased activity in erectile dysfunction. This pathway helps maintain penile vascular homeostasis and is regulated by the tonic release of NO in smooth muscle cells. NO/cGMP kinase promotes RhoA expression, enhancing transcription and protein stability. Studies show that Rho-kinase activity is reduced in the penile tissue of eNOS-deleted mice but can be corrected with eNOS gene transfer. In priapism, deficient endothelial NO disrupts this feedback mechanism, downregulating RhoA/Rho-kinase activity. This, along with a lack of cGMP regulation, reduces cavernosal smooth muscle tone and leads to an exaggerated response to erectogenic stimuli [20–22]. 

On the other hand, Adenosine is known to be an effective vasodilator and regulator of penile tumescence. It works by increasing cyclic adenosine monophosphate (cAMP) and activating protein kinase A, leading to decreased intracellular calcium and SMC relaxation. Adenosine also boosts NO release from endothelial cells, enhancing SMC relaxation. Elevated adenosine activity, triggered by cellular damage, hypoxia, and ischemia, is linked to priapism. Studies show significantly higher adenosine levels in mouse models with recurrent priapism and its association with penile fibrosis. These findings suggest a key role for adenosine in the pathophysiology of priapism [23–25]. 

Another mediator, called opiorphin, belongs to a unique family of peptides and was recently described to be involved in priapism-like outcomes by influencing penile cavernosal smooth muscle tone. Tong et al. have demonstrated that increased corporal smooth muscle relaxation can be achieved following overexpression of the genes encoding opiorphins. The mechanism of action of opiorphins counts on the potent inhibition of neutral endopeptidases (NEP) which is responsible for the metabolism of multiple signaling peptides. By inhibiting NEP, opiorphins allow a prolonged binding of peptide agonists to receptors, thereby promoting the relaxation of corporal smooth muscles [26, 27]. 

Other signaling molecules, including heme oxygenase, carbon monoxide, androgens, alpha-adrenergic agonists, and transforming growth factor beta, also play roles in the pathophysiology of priapism, although their mechanisms are less well understood [13, 28–30]. 

## Result analysis

The literature review will be divided into two parts to comprehensively address the subject. The first part will focus on the evaluation and management of stuttering priapism patients, covering diagnostic approaches, patient assessment, and clinical considerations. The second part will delve into the treatment options available for priapism, exploring various therapeutic interventions, their effectiveness, and potential outcomes.

### Evaluation and diagnosis

#### 1- History taking

A complete medical history focused on the circumstances of onset, duration, and possible risk factors are of paramount value. Systemic medical conditions such as hematological dyscrasias and malignancies, drug history such as self-injection of intracavernosal agents for ED or recreational sex and the use of antipsychotics, a previous history of genito-urethral trauma to rule out high flow priapism and the presence of a prior history of priapism should all be taken into consideration in the case of idiopathic stuttering priapism [31]. Clinically, these priapic events can present as non-resolving morning erection or sleep-related in 77% of the cases, or be associated with sexual stimulation in 17% of the cases. The degree of pain must be assessed to differentiate between ischemic priapism, which is always painful, and high-flow non-ischemic priapism which is not painful. The progressive nature of penile pain with the duration of the erection characterizes ischemic priapism. Treatment should be instigated within 4 h to avoid irreversible corporal fibrosis. Usually, the mean duration of priapic events is around 125 min with a peak incidence around 3:00 a.m [13, 32]. A focused sexual history before priapism must be taken to understand the baseline erectile function and sexual performance, as well as to determine the expected level of function after treatment. This assessment can be accurately performed using patient-reported questionnaires such as the Priapism Impact Profile and the International Index of Erectile Function [33, 34]. 

### 2- Clinical examination

The physical examination may reveal some signs to differentiate between ischemic and non-ischemic priapism. Usually in ischemic priapism, the corpora cavernosa is engorged, fully rigid, and painful. Sometimes, the glans penis and corpus spongiosum are found to be engorged as well and this is considered to be a sign of corporal infarction. Nevertheless, in non-ischemic high-flow priapism, the corpora cavernosa, although tumescent, are neither fully rigid nor painful [13, 31]. In ISP, the examination of the lower abdomen, penis, testicles, perineum, rectum, and prostate is mandatory to rule out other causes or other types of priapism. There is always a possibility to uncover a tumor, a mass, or a site of infection which are deemed rare but relevant causes of priapism. On the other hand, penile examination may reveal for example an injection site hindered as a cause of priapism [13, 35, 36]. Sometimes, the Piesis sign, described as the complete penile detumescence after the application of pressure to the perineum, is used to rule out arterial priapism and is most commonly seen in children [37]. 

### 3- Laboratory analysis

Laboratory testing is essential and includes a complete blood count, white blood count with differential, platelet count, hemoglobin, coagulation profile, and serum biochemistry profile to identify hematological dyscrasias. Specific tests like a sickle cell screen may be conducted based on patient age and race due to the ethnicity-related prevalence of certain disorders [12, 13]. If there is suspicion of recreational drug use, both urine and plasma toxicology screens should be performed, along with blood tests for psychoactive medication levels, especially in patients with a psychosocial history [13, 38–40]. Assessing the hypothalamic-pituitary-gonadal axis hormonal function by measuring serum testosterone may also be recommended [41]. If stuttering episodes progress to acute ischemic priapism, emergency corporeal aspiration with blood gas evaluation is needed for both diagnosis and treatment. Ischemic priapism blood appears dark, with pO2 < 30 mmHg, pCO2 > 60 mmHg, and pH < 7.25, while non-ischemic blood is bright red [31, 42]. 

### 4- Imaging

Color duplex ultrasonography (CDU) can play a role in the differentiation between ischemic and nonischemic priapism in association with patient history, clinical examination, and blood gas analysis, the importance of CDU in ischemic priapisms lies in its ability to detect high resistance and low velocity cavernosal arterial flow [7, 43]. Whereas the CDU appearance of stuttering priapisms is similar to that of a normal erection. Usually, a high and variable velocity flow is documented in the cavernosal arteries reaching 30–40 cm/s or more with diastolic flow disappearance or reversal and low peripheral resistance. With a rigid erection state, peak systolic velocities reduce [7, 34]. This could be the sonographic manifestation of a reduced, fluctuating smooth muscle tone as discussed by Patel et al. [44] The reduction or disappearance of cavernosal artery flows is a significant sign that a major ischemic priapic episode has been installed and that emergent treatment is compulsory [43]. The role of Magnetic Resonance Imaging (MRI) in this setting depends on the type of priapism. In ischemic priapism, it clarifies the degree of corporal infarction which may influence the therapeutic approaches applied. Whereas, in high-flow non-ischemic priapism, dynamic post-contrast images may detect a fistulous origin. However, MRI seems to have no useful role in the treatment decisions for stuttering priapism [45]. Arteriography has no role in stuttering priapism. However, it is employed precisely to identify arterial fistulae in non-ischemic priapism to selectively embolize the damaged vessel. If clinically indicated, CT scans can be electively done to detect a suspected mass or malignancy [38]. 

### Treatment approaches

In treating ischemic and non-ischemic priapism, differentiation between the two is paramount. Conservative measures are typically the first line for non-ischemic cases, with embolization or surgery reserved for severe or refractory instances. Ischemic priapism treatment options range from medical interventions like aspiration and injections to minimally invasive procedures and surgery, all aimed at preventing erectile tissue damage [7]. Stuttering priapism poses a particular challenge due to its complex etiologies, leading to a lack of standardized treatment approaches. While systemic therapies play a role in recurrence prevention, pharmacologic agents targeting underlying pathophysiology lack consistent efficacy. The primary objective across all priapism types is preventing recurrence and progression to irreversible corporal fibrosis, emphasizing systemic and minimally invasive treatment modalities to halt ischemic changes [46, 47]. 

#### 1- Patient counseling and conservative treatment

Patient counseling aims to provide the patient suffering from these recurrent priapic episodes with sufficient information regarding the natural course of the disease and the probable associated risk factors. Initial supportive measures that can be applied in the acute setting at home and the possible ways to seek medical advice, if needed, should be transmitted to the patient, the goal of which is to reduce the patient’s anxiety, and alleviate the progression to acute ischemic attacks [48]. Counseling also takes into consideration the knowledge of the patient about the medications with a priapism as a potential side effect such as alpha-adrenergic receptor blockers and the full recognition of a disease with a high risk of priapism like hematological dyscrasias and tumors [49]. 

Historically, several practical conservative methods were initially applied by the patient; they involved analgesia, opioid use, ejaculation, physical exercise, fluid intake, cold baths, urination, ejaculation, cold water enemas, or cold packs. The rationale behind these maneuvers relies on the increase of the heart rate and venous return or the stimulation of the noradrenergic system subsequent detumescence [7, 13, 31, 50, 51]. Unfortunately, all these conservative tactics lack substantial evidence of efficacy and there is no considerable data in the literature to prove their effectiveness [31, 52, 53] However, their use can still be applied as long as there is no delay in appropriate management [54]. Also, counseling the patient about the impact of stuttering priapism on his future sexual life helps in lessening the influence of this disorder on his mental health and adjusts his social relationships [48]. 

#### 2- Treatment of an acute episode

Given that ISP may evolve into acute ischemic events, urologists and primary care physicians should be familiar with all practical solutions that decrease the risk of penile compartment syndrome. After failure of conservative approaches, the standard of care is corporal blood aspiration with or without irrigation in combination with alpha agonists instillation directly into the corpus cavernosum. The main goal of this procedure is to preserve the SMC by eliminating the cavernosal blood clots and allowing new fresh oxygenated arterial blood to flow in the corpus cavernosum. An 18- or 19-gauge Angio catheter or a butterfly needle is used at the base of the penis in the 3 o’clock and/or 9 o’clock position. A maneuver of irrigation of the corpora with a saline solution 0.9% can break down the clots and enable as such the aspiration of blood clots. The use of cold saline at 10 degrees Celsius during irrigation improves the rate of complete detumescence. More importantly, aspiration should be continued until obtaining a brighter color of the aspirated blood. Multiple attempts of this first-line maneuver should be repeated several hours before confirming the resolution of the acute episode. Another determinant of success is the resolution of pain and penile detumescence. Some clinicians use two needles placed at the same time at the base of the penis to inject, irrigate and aspirate concurrently [55]. The literature puts the rate of success of this maneuver at around 36% [6, 7, 31, 56]. If ineffective, and within 1–2 h of a maintained erection, the injection of alpha agonists directly into the corpus cavernosum becomes very valuable [57]. 

Phenylephrine is the drug of choice since it is very selective for the alpha1-adrenergic receptor with no activity on the beta-adrenergic allowing it to be denuded from any beta-mediated cardiac inotropic or chronotropic side-effects. The appropriate concentration is 100–500 µg/mL after diluting phenylephrine in normal saline. The usual dose of each injection is 200 µg every three to five minutes with a maximum of 1 mg within one hour. Blood pressure and pulse rate should be closely observed every 15 min for an hour after the injections [58, 59]. 

The resulting hypoxia, acidosis, and glucopenia induced by priapism, generate a decline in α-receptor affinity. This may justify the occasional failure of α-adrenergic agonists to alleviate prolonged erections by inefficiently stimulating corporal smooth muscle contraction [28]. The rate of success and detumescence after sympathomimetic intracavernosal injection use increases up to 80% [6, 31]. Other sympathomimetic agents used are, but not limited to: etilefrine: 2.5 mg diluted in 1–2 mL saline, adrenaline: 2 mL of 1/100,000 solution given up to 5 times in 20 min, methylene blue: 50–100 mg intracavernosal injection followed by aspiration and compression. However, until now there is no published direct efficacy comparison between these α-agonists [31]. When in doubt, the treating physician should reevaluate the current state of the disease by the examination of corporal rigidity, the assessment of cavernous blood gas testing for acidosis and anoxia, checking the absence of cavernosal artery inflow by penile CDU or the verification of elevated intracorporal pressure by pressure monitoring [6]. 

Methylene blue use was described in the literature in the acute setting of priapism. It is given as an injection of 10 cc at a concentration of 10 g/mL. Its role consists of inhibiting endothelial-mediated cavernosal relaxation that causes temporary control of symptoms with the heightened risk of penile burning [5, 60]. 

#### 3- Prevention of recurrence

Patients with recurrent ischemic priapism should be seen in a clinic setting after initial management with an early follow-up appointment to explain the natural course of their disease, ascertain any associated risk factors, and avoid any recurrent event without an insured adequate treatment.

Recurrent episodes of priapism may carry an elevated risk of complications. Consequently, the goal in managing patients with stuttering priapism would be to strategically prevent future episodes, preferably using drugs that combine a safe profile, good tolerability, and high efficiency.

Intracavernous treatments for intermittent stuttering priapism primarily involve injecting alpha-adrenergic agonists, such as phenylephrine, into the corpora cavernosa to induce detumescence by promoting vascular contraction. These agents, including metaraminol, ephedrine, etilefrine, epinephrine, norepinephrine, and phenylephrine, have been effective in resolving acute ischemic episodes. Comparative efficacy data show detumescence in 81% of cases treated with epinephrine, 70% with metaraminol, 65% with phenylephrine, and 43% with norepinephrine. However, the literature lacks an efficacy comparison between these α-agonists [7, 41, 61]. The importance of these agents is the possibility of self-injections in cases of emergency [62]. Self-injection protocols for alpha-agonists can reduce emergency visits, though data on outcomes are limited. These treatments are contraindicated in patients with malignant hypertension or those on monoamine oxidase inhibitors. Proper patient education on injection techniques, dosage, and adherence is crucial [31]. Monitoring for adverse effects, such as hypertension, arrhythmias, and local reactions, is recommended, especially in high-risk patients. Rare severe complications include subarachnoid hemorrhage and myocardial infarction [63, 64]. 

Alternative treatments like tissue plasminogen activator (TPA) and methylene blue (MB) have shown some efficacy in specific cases but lack widespread evidence for routine use. TPA is not recommended for self-administration due to bleeding risks [65]. While no drug-related adverse effects are reported for MB injections, its effectiveness in stuttering priapism remains unsubstantiated [41, 66]. 

Concerning oral treatment, and especially hormonal treatment for stuttering priapism aims to suppress circulating testosterone to hypogonadal levels using various agents. These include GnRH agonists or antagonists, finasteride, diethylstilbestrol (DES), ketoconazole, and antiandrogens, all targeting different mechanisms of testosterone regulation. Reducing testosterone levels can decrease libido, reduce nocturnal erections, and induce erectile dysfunction by altering penile tissue at the molecular level, affecting NOS isoforms, PDE-5, and SMC differentiation, among other pathways [67, 68]. However, androgen suppression has significant drawbacks, such as impaired sexual function, pubertal growth issues, and metabolic and cardiovascular adverse effects. These side effects necessitate caution, especially in prepubertal patients and those desiring fertility. Despite limited literature on hormonal treatments for priapism, anti-androgens and GnRH agonists are considered effective first-line treatments, although the optimal duration of therapy remains unclear [29, 46, 69]. 

GnRH agonists, notably Goserelin Acetate and Leuprolide, initially increase LH production, followed by receptor desensitization and a decrease in LH/FSH production, reducing testosterone levels. Typically administered as 7.5 mg intramuscular injections monthly, they effectively prevent priapism episodes but can cause hot flashes, gynecomastia, loss of libido, erectile dysfunction, and osteoporosis. Despite these side effects, many patients retain sexual activity capability [70, 71]. 

Ketoconazole, typically used as an antifungal agent, inhibits adrenal and testicular steroid production by blocking cytochrome P450 14-alpha-demethylase [20]. Its side effect of reducing testosterone production has been leveraged for treating stuttering priapism. Standard treatment involves 200 mg of ketoconazole three times daily with prednisone for two weeks, followed by a maintenance dose. While effective in symptom resolution, ketoconazole can cause GI upset, gynecomastia, QT prolongation, and hepatotoxicity. Importantly, it preserves sexual function, making it suitable for prepubertal men and those wishing to maintain fertility [72–74]. 

Antiandrogen agents like flutamide, bicalutamide, and chlormadinone effectively suppress nocturnal erections by blocking androgen receptors, preventing testosterone and dihydrotestosterone from binding. Unlike GnRH agonists and estrogens, antiandrogens maintain libido, a significant advantage [74, 75]. Flutamide (250 mg thrice daily), bicalutamide (50 mg daily), and chlormadinone (100 mg daily) are common regimens, though optimal treatment duration is unclear. Side effects include gynecomastia, hot flashes, osteoporosis, and diarrhea, with higher doses potentially causing erectile dysfunction. Antiandrogens offer a favorable safety profile, and case reports indicate their success in preventing priapism, especially when combined with alpha-adrenergic agonists. Further research is needed to establish long-term efficacy and safety [20, 75–77]. 

Diethylstilbestrol and ethinylestradiol, synthetic estrogens, inhibit the pituitary gland, reducing male hormone levels and potentially reversing endothelial-mediated processes regulating detumescence [78, 79]. DES (5 mg daily for 2–4 weeks) and low-dose ethinylestradiol (25 mg at bedtime) have shown effectiveness in short-term priapism treatment, though high relapse rates upon discontinuation are noted. Side effects include loss of libido, erectile dysfunction, gynecomastia, cardiovascular incidents, osteoporosis, and thromboembolic events. Estrogen therapy’s risks necessitate thorough patient counseling, and long-term use is generally not recommended [80, 81]. 

5-Alpha Reductase Inhibitors, such as finasteride and dutasteride, inhibit the enzyme 5-alpha-reductase, reducing the conversion of testosterone to DHT [82]. These agents, used primarily for benign prostatic hyperplasia (BPH) and hair loss, have shown promise in preventing idiopathic stuttering priapism. Finasteride (initially 5 mg daily, tapered over time) and dutasteride (0.5 mg daily or tapered) have demonstrated efficacy in symptom resolution within 3–4 months. Side effects are rare but include gynecomastia, fatigue, and sexual issues. Long-term studies indicate significant symptom improvement and reduced emergency room visits, making 5ARIs a promising treatment option, though further validation is required [83]. 

Recent preclinical studies suggest hypogonadism as a risk factor for recurrent priapic episodes [84]. Testosterone replacement therapy in hypogonadal men may reduce the incidence of these episodes by restoring eNOS/cGMP activity and PDE5 expression to normal levels. Studies by Goglia et al., Morrison et al., and Burnett et al. indicate that TRT is safe and can improve sexual function without increasing priapism frequency. However, careful monitoring is necessary to avoid supra-physiological testosterone levels, which may worsen priapism by inhibiting eNOS activation and exacerbating molecular derangements [85–87]. 

Digoxin, commonly used for congestive heart disease, has been investigated for stuttering priapism due to its ability to regulate smooth muscle tone by increasing intracellular calcium levels [88]. Gupta et al. found that digoxin decreases sexual desire and penile rigidity, aiding detumescence without affecting libido or hormone levels [89]. However, digoxin’s narrow therapeutic index and side effects necessitate blood-level monitoring, making it a secondary treatment option.

Baclofen, a GABA analog, is used to treat spasticity and reflexogenic erections in patients with spinal cord injuries [90]. Intrathecal baclofen has shown efficacy in reducing penile erection rigidity and preventing reflexogenic erections, although the exact mechanism remains unclear. Oral baclofen appears less effective, but some reports suggest it can reduce the frequency and duration of stuttering priapism episodes. The recommended dose is 10 mg nightly, increased up to 30 mg per day. Side effects include nausea, confusion, drowsiness, fatigue, headache, and hypotension. More studies are needed to clarify baclofen’s role in stuttering priapism and its distinction from reflexogenic erections [91, 92]. 

Gabapentin, a voltage-gated calcium channel inhibitor, is widely used as an anticonvulsant, antinociceptive, and anxiolytic drug. Its reported sexual dysfunction, such as anorgasmia and decreased potency, forms the basis for its use in treating stuttering priapism. Gabapentin inhibits Ca2 + influx by blocking voltage-operated Ca2 + channels, leading to reduced smooth muscle relaxation in the corpora. Additionally, studies in rats have shown that gabapentin reduces testosterone and follicle-stimulating hormone levels, potentially contributing to its effectiveness in treating priapism. Despite its apparent efficacy, further studies are necessary to confirm whether patients can maintain normal sexual function and to elucidate the precise mechanisms of action. The recommended dose is 0.3–0.8 g three times per day [93, 94]. 

Terbutaline, a β2-adrenergic receptor agonist with some α-agonist properties, promotes smooth muscle relaxation and is promising for preventing priapic relapses in sickle cell disease (SCD) patients by increasing arterial blood flow and flushing out stagnant sickle cells. Both oral and subcutaneous forms have been shown to reduce the frequency and duration of priapic events. The recommended dose is 5 mg orally, increasing to 10 mg if the erection persists after 30 min. Side effects include headache, flushing, diaphoresis, tachycardia, and nausea. Terbutaline is effective for pharmacologically induced priapism but not recommended for idiopathic stuttering priapism due to limited studies on its effectiveness in this context. It is contraindicated in patients with diabetes, hypertension, hyperthyroidism, and seizures [95–97]. 

PDE5 inhibitors like sildenafil and tadalafil, traditionally used for erectile dysfunction, have shown promise in treating stuttering priapism through a paradoxical effect [98]. By maintaining steady-state cGMP levels in corporal tissues, PDE5 inhibitors normalize PDE5 expression and activity, correcting molecular dysregulation. Chronic low-dose PDE5 inhibitor therapy increases phosphorylated eNOS protein expression, restores NO production, and alleviates oxidative stress, reducing priapic episodes [99, 100]. Studies have demonstrated reduced ER visits and episode frequency with daily tadalafil (5 mg/day) [101]. PDE5 inhibitors are generally well-tolerated, with minimal side effects. The recommended regimen includes sildenafil 25 mg daily or tadalafil 5 mg daily or every other day, with efficacy observed within 2–4 weeks. Patients should avoid nocturnal dosing and sexual stimulation shortly after ingestion to prevent sexually-associated priapism events [102, 103]. 

Salbutamol, a β2-agonist with some β1 and α-agonist activity, promotes penile detumescence through mechanisms like smooth muscle relaxation and increased venous drainage. Although its use in stuttering priapism is scarcely documented, case reports suggest potential benefits. More studies are needed to confirm its effectiveness and establish a clear mechanism of action [59, 104]. 

Hydroxyurea, an antineoplastic agent, inhibits DNA synthesis by binding to ribonucleotide reductase enzymes. It is used for hematological neoplasms and shows promise in treating SCD-associated stuttering priapism by increasing NO production and restoring NO/cGMP pathway equilibrium. The recommended dose is 10–35 mg/kg/day, adjusted based on CBC (Complete Blood Count) monitoring. Higher doses may be needed to reduce priapic episodes. Hydroxyurea’s impact on male fertility, particularly oligospermia, is a concern. Despite its benefits in SCD-related priapism, more data is needed for its role in idiopathic stuttering priapism [105–107]. 

Oral sympathomimetic agents like pseudoephedrine are not recommended for major ischemic priapism but have been used to prevent recurrent episodes. The Priapism in Sickle Cell Study (PISCES) suggests their utility is still uncertain, requiring further investigation. Pseudoephedrine (30–60 mg daily) has shown some efficacy, with no significant adverse effects on hypertension or sexual function. However, large-scale studies are needed to assess their prophylactic effects comprehensively [108, 109]. 

On the other hand, surgeons may resort to surgical interventions as a solution to ISP.

When an acute priapic episode transforms into a major ischemic event and if all conservative managements fail including repeated cavernosal blood aspiration and injections of alfa-adrenergic agonists or if the patient develops significant cardiovascular events secondary to intracavernosal injection therapy with sympathomimetics in the acute setting, surgery would be deemed necessary as a second line therapy to decompress the corporeal bodies [13]. 

However, the amount of time required to move to the second-line therapy is still to be defined. It has been revealed that conservative measures including aspiration with or without intracorporeal instillation of a-adrenergic agonists are acceptable and successful in the initial stages of priapism (< 24–36 h). If the duration of priapism exceeds 48 h, the shunt surgery would be less successful [31]. 

The principle of the shunt surgery relies on the diversion of the ischemic blood from the corpus cavernosum into the corpus spongiosum or the venous system by creating an opening in the corpora cavernosa to reinstate a normal circulation and subsequently alleviate penile ischemia and prevent fibrosis.

Distal shunts are recommended to be challenged first, by both the EAU and the AUA guidelines, before considering proximal shunts as the former is associated with less risk of complications and is technically easier. Multiple types of distal shunts are described. The Winter’s shunt, the Ebbehoj’s shunt, and the T-shunt are all considered techniques of percutaneous distal corpora-glandular shunts in which the utilization of a Tru-Cut^®^ biopsy needle or scalpel creates a fistula between the corpus spongiosum and the corpora cavernosa. Open surgery is an alternative to percutaneous shunts. Winter shunt is the most common shunting procedure practiced nowadays owing to the rapidity and the success rate of the procedure approaching 50 to 65% of the cases. However, this technique carries a substantial risk of recurrence and leads to erectile dysfunction when it is carried out within 24 h of the priapism onset. On the other hand, preservation of erectile function is observed if the procedure is carried out after 24 h of the onset of the episode [7, 110, 111]. During shunting procedures, it is interesting to take a cavernosal smooth muscle biopsy as this will help counsel the patient and model the decision-making regarding the need for further implant surgery [112, 113]. 

Penile prosthesis surgery is a last resort option in the treatment of ISP because of its irreversible nature. It should be reserved for patients with majorly impaired quality of life and for those with concomitant disturbed erectile function that precludes adequate sexual practice. It is also a fact that when priapism lasts more than 24 h, more than 90% will fall in the category of erectile dysfunction [114]. 

Early versus delayed penile prosthesis surgery has always been a matter of debate. The EAU committee states that penile prosthesis, regardless of the type, should be considered at the time of presentation if the ischemic episode has been present for more than 36 h and only after failed conservative management including aspiration and sympathomimetic intracavernous injections and futile shunting procedures since it is considered as a permanent intervention that impends natural sexual function [6, 31]. 

Several benefits of an early implantation grab the attention of clinicians including, the absence of corporal fibrosis that complicates and compromises penile prosthesis insertion, the reliability in penile length preservation, the prevention of penile deformity, the earlier return of sexual activity, the ease of technical feasibility and the higher satisfaction rate. On the other hand, it is associated with a substantial risk of infection and distal erosion, especially if the patient was previously treated with distal shunt operations. The problem that occurs in early penile prosthesis implantation is the protrusion of the distal cylinder through a defect in the corpora second to previous shunt surgery. To bypass this issue, Salem et al. proposed an easy and simple method by which they attach the cylinder to the tunica albuginea by a non-absorbable sling suture. When the tunica is intact after shunting, the risk of device erosion is tremendously reduced [113–115]. 

Penile implantation as a delayed procedure could have technical challenges due to scarring and fibrosis and often requires the use of downsized shorter cylinders [56]. So penile prosthesis insertion should preferably be done at the time or shortly after the corporal dilation, ultimately within not more than a few weeks. In general, studies have shown that the risk of failure with penile prosthesis implantation in the context of priapism is higher than for elective cases [116]. As the implantation penile prosthesis is considered a definitive surgery, this treatment option should be reserved for patients suffering from disturbed sexual function and subsequent poor quality of life or those having concomitant erectile dysfunction impairing sexuality [69]. 

Another technique is subcapsular orchidectomy. Although outdated in the presence of GnRH agonists, can be applied in cases of stuttering priapism refractory to oral therapy for patients with relative contraindications or with intolerable side effects of medical treatment [88]. 

### Emergent therapies

Emerging research on the molecular pathways involved in stuttering priapism has led to the development of new prophylactic strategies. Oxidative Stress Regulators play a significant role in managing stuttering priapism by targeting the underlying mechanisms of disease onset and progression.

Apocynin: This oxidative stress inhibitor, which acts by inhibiting NADPH oxidase, has shown promise in increasing basal NO levels, a critical factor in the pathophysiology of stuttering priapism [8]. 

Theophylline: Known as an adenosine receptor antagonist, theophylline has been demonstrated to counteract priapism induced by excessive adenosine, a substance with vasodilatory effects similar to NO. ADA enzyme replacement therapy is a potential approach to reduce elevated adenosine levels and its prolonged effects on penile vasodilation [117, 118]. 

Opiorphins: These peptides, which play a role in erectile function, have shown increased expression in models of sickle cell disease. Targeting the polyamine synthetic pathway, particularly the enzyme ornithine decarboxylase (ODC), could provide a novel therapeutic approach for stuttering priapism [119]. 

Other Agents: Hydralazine and procyclidine have also been identified as potential treatments for stuttering priapism. Further research is needed to confirm their efficacy and clinical applicability [120]. 

These advancements offer new insights and potential treatments for managing stuttering priapism, although additional studies are necessary to validate these approaches in clinical practice.

## Conclusion: the algorithmic approach

Erectile function is endangered in each acute priapic event as it can progress to a major classic ischemic priapism with subsequent irreversible corporal fibrosis. Thence, to avoid these complications, the prevention of recurrence is the main objective in this scenario.

As the etiologies of idiopathic stuttering priapism are diversified, the treatment approach is not standardized. Systemic therapies play a significant role in the prevention of recurrence. However, available pharmacologic agents aiming at correcting the underlying pathophysiology fail to show a steady result in thwarting recurrences. The prevention of priapic events progression into major ischemic attack and a true compartment syndrome is based on systemic as well as minimally invasive treatment modalities and is headed toward stopping ischemic changes.

**Fig. 2:**
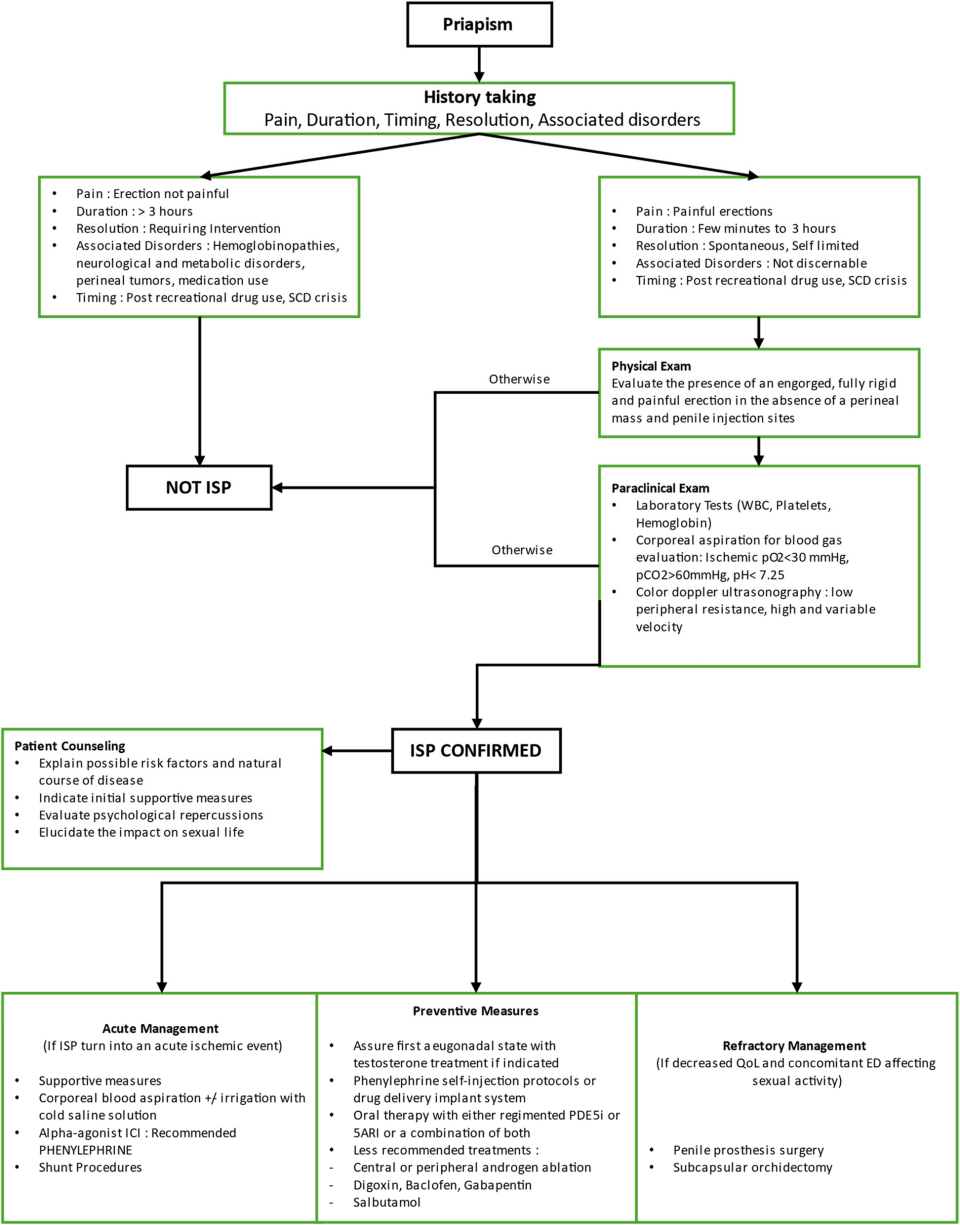
Proposed algorithm for the evaluation and management of ISP. Legend: This proposed algorithm provides a structured approach for the evaluation and management of Intermittent Stuttering Priapism (ISP). The process involves the following steps: 1. History Taking: Primarily used to identify ISP, with the aid of physical examination and paraclinical tests to confirm the diagnosis. 2. Patient Counseling: Once ISP is confirmed, provide patient counseling to discuss the condition and its implications. 3. Management Categories: - Acute Management: Immediate interventions to manage active priapic episodes. - Preventive Measures: Strategies to reduce the frequency and severity of future episodes. - Refractory ISP Management: Advanced treatments for cases that do not respond to standard management approaches. Abbreviation List for the figure : 1. 5 ARI - 5-Alpha Reductase Inhibitors. 2. ED - Erectile Dysfunction. 3. ISP - Intermittent Stuttering Priapism 4. PDE5i - Phosphodiesterase Type 5 Inhibitors. 5. QoL - Quality of Life . 6. WBC - White Blood Cell

To this end, we propose a handy tool for clinicians in the treatment framework of ISP to facilitate clinicians’ access to the literature without extensive return to case reports and review studies (Fig. 2).

## Data Availability

Data and materials used or analyzed in this study can be made available by contacting the corresponding author upon a reasonable request.

## Abbreviations

5ARI − 5: Alpha Reductase Inhibitors
AUA: American Urological Association
BPH: Benign Prostatic Hyperplasia
CBC: Complete Blood Count
CDU: Color Doppler Ultrasound
cAMP: Cyclic Adenosine Monophosphate
cGMP: Cyclic Guanosine Monophosphate
CT: Computed Tomography
DES: Diethylstilbestrol
DHT: Dihydrotestosterone
EAU: European Association of Urology
ED: Erectile Dysfunction
ER: Emergency Room
FSH: Follicle-Stimulating Hormone
GABA: Gamma-Aminobutyric Acid
GI: Gastrointestinal
GnRH: Gonadotropin-Releasing Hormone
ISP: Idiopathic Stuttering Priapism
LH: Luteinizing Hormone
MRI: Magnetic Resonance Imaging
NADPH: Nicotinamide Adenine Dinucleotide Phosphate
NEP: Neprilysin
NO: Nitric Oxide
NOS: Nitric Oxide Synthase
ODC: Ornithine decarboxylase
pCO2: Partial Pressure of Carbon Dioxide
pH: Power of Hydrogen (measure of acidity or alkalinity)
PKG: Protein Kinase G
pO2: Partial Pressure of Oxygen
PDE5: Phosphodiesterase Type 5
PRISMA: Preferred Reporting Items for Systematic Reviews and Meta-Analyses
SCD: Sickle Cell Disease
SMC: Smooth Muscle Cells

## Data availability

No datasets were generated or analysed during the current study.
